# Perovskite Solar Cells Yielding Reproducible Photovoltage of 1.20 V

**DOI:** 10.34133/2019/8474698

**Published:** 2019-03-18

**Authors:** Essa A. Alharbi, M. Ibrahim Dar, Neha Arora, Mohammad Hayal Alotaibi, Yahya A. Alzhrani, Pankaj Yadav, Wolfgang Tress, Ahmed Alyamani, Abdulrahman Albadri, Shaik M. Zakeeruddin, Michael Grätzel

**Affiliations:** ^1^Laboratory of Photonics and Interfaces, Institute of Chemical Sciences and Engineering, École Polytechnique Fédérale de Lausanne, Lausanne CH-1015, Switzerland; ^2^National Nanotechnology Research Centre, King Abdulaziz City for Science and Technology (KACST), P.O. Box 6086, Riyadh 11442, Saudi Arabia; ^3^National Center for Petrochemicals Technology, King Abdulaziz City for Science and Technology (KACST), P.O. Box 6086, 11442 Riyadh, Saudi Arabia

## Abstract

High photovoltages and power conversion efficiencies of perovskite solar cells (PSCs) can be realized by controlling the undesired nonradiative charge carrier recombination. Here, we introduce a judicious amount of guanidinium iodide into mixed-cation and mixed-halide perovskite films to suppress the parasitic charge carrier recombination, which enabled the fabrication of >20% efficient and operationally stable PSCs yielding reproducible photovoltage as high as 1.20 V. By introducing guanidinium iodide into the perovskite precursor solution, the bandgap of the resulting absorber material changed minimally; however, the nonradiative recombination diminished considerably as revealed by time-resolved photoluminescence and electroluminescence studies. Furthermore, using capacitance-frequency measurements, we were able to correlate the hysteresis features exhibited by the PSCs with interfacial charge accumulation. This study opens up a path to realize new record efficiencies for PSCs based on guanidinium iodide doped perovskite films.

## 1. Introduction

Over the past few years, perovskite solar cells (PSCs) have attracted great attention in the applied and theoretical research fields [1]. The appealing photophysical properties, such as small exciton binding energy, tunable bandgap, high absorption coefficient, relatively high carrier mobility and diffusivity, and tolerance to defects, broaden the application horizon of perovskites from photovoltaics to light emitting diodes and lasing [2–6]. Since PSCs were first introduced by the Miyasaka group in 2009, the power conversion efficiency (PCE) has been improved from 3.8% to over 23% using solution-based deposition methods [2, 7]. However, these methods can generate films exhibiting pinholes and defects, which are detrimental to the device performance due to the occurrence of parasitic charge carrier recombination under operational conditions [8, 9]. Therefore, to develop high-efficiency PSCs using solution-based approaches, one of the challenges is to achieve a rational control over the quality of the absorber layer [10, 11]. Recent reports on PSCs have shown that nonradiative charge carrier recombination or charge injection at the interfaces to charge transport layers limits the open circuit voltage (*V*_OC_) [12–15]. The role of grain boundaries acting as recombination centers is under debate as such processes depend on the processing conditions and composition of the perovskite layer [16–20].

As it has become important to suppress the nonradiative recombination losses occurring at the interfaces or throughout the bulk of the perovskite film [21], many efforts have been devoted towards passivating the defects, which consequently retard the trap-assisted nonradiative charge carrier recombination. For example, a series of dialkylammonium and phenyltrimethylammonium halides were successfully explored to passivate the interfaces and grain boundaries resulting in a much-improved PCE [22–24]. In a similar manner, the guanidinium (Gua) cation (CH_6_N_3_^+^) was employed for the passivation of nonradiative recombination centers at CH_3_NH_3_PbI_3_ grain boundaries enhancing the *V*_OC_ up to 1.07 V [25–27]. The Gua cation may also act as a cross-linker between the neighboring perovskite grains in the film [28]. It is worth emphasizing that the Gua cation was mainly investigated as an additive to CH_3_NH_3_PbI_3_ based PSCs displaying moderate power conversion efficiencies. In addition to forming mixed-cation phases exhibiting relatively large bandgap, Gua cation can mix with MA and/or FA cations in the 3D perovskite lattice [29, 30] and consequently influence the excitonic and structural properties of the host phase remarkably. However, its influence on the bandgap, film quality, and performance of multication-based PSCs has not been explored so far.

In this article, we illustrate the effect of guanidinium iodide (GuaI) on the photophysical properties of mixed-cation and mixed-halide perovskite films. Thorough analyses based on scanning electron microscopy and emission studies were carried out to investigate the effect of GuaI addition on the morphology and emission of the perovskite material. Furthermore, electroluminescence and time-resolved photoluminescence studies were performed, respectively, to investigate the fully assembled devices and the charge carrier recombination occurring within the perovskite films. The operational stability carried out at a maximum power point under continuous full-sun illumination for 200 h showed that the PSCs containing Gua cations are as stable as control PSCs. In addition to providing new physical insights, we demonstrate for the first time robust stability for Gua based PSCs which are >20% efficient and yield photovoltage as high as 1.20 V.

## 2. Results

As a reference PSC, we used the mixed-halide and mixed-cation formulation “Cs_0.05_(MA_0.17_ FA_0.83_)_0.95_Pb(I_0.83_ Br_0.17_)_3_” (abbreviated as Cs_5_Pb). By adding* x* = 5% volume ratio of 1.5 M GuaI in DMSO, we prepared Cs_5_Pb·*5*GuaI perovskite films [31].

### 2.1. Steady-State and Time-Resolved Photoluminescence

**Figure 1:**
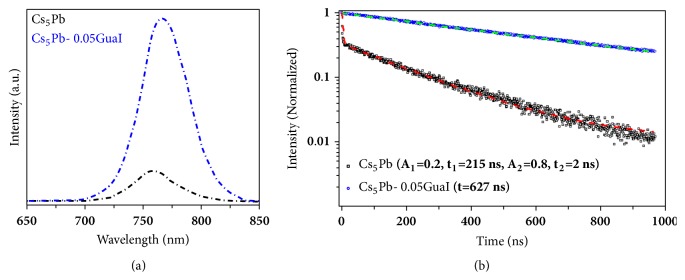
Photoluminescence studies of pure and GuaI containing perovskite films. (a) Steady-state photoluminescence, (b) time-resolved photoluminescence spectra; solid lines present the fitting curves obtained using a mono- or biexponential decay model.

To unravel the impact of GuaI on the emission properties of the absorber layer, steady-state photoluminescence (PL) was measured for Cs_5_Pb.*5*GuaI films (Figure 1(a)). Upon addition of 5% GuaI, a slight red shift from 757 to 766 nm in the peak position indicates that incorporation of GuaI in the perovskite lattice decreases the band gap slightly. In addition, the emission intensity enhanced dramatically with the incorporation of GuaI in Cs_5_Pb precursor solution indicating a decrease in the nonradiative recombination.

Variation in the steady-state PL intensity apparently demonstrates that the level of defects promoting nonradiative carrier recombination in the perovskite film decreases upon introducing GuaI into the precursor solution. We employed time-resolved photoluminescence (TRPL) to further characterize the perovskite films quantitatively (Figure 1(b)). While exciting the films from the perovskite side using 408 nm photons, we studied the charge carrier dynamics occurring within the reference and Cs_5_Pb.*x*GuaI perovskite films. Figure 1(b) shows that the incorporation of 5% GuaI dramatically increases the charge carrier lifetime. Charge carrier lifetime of 215 ns and 627 ns was estimated, respectively, for the Cs_5_Pb, and Cs_5_Pb.0.05GuaI films. The initial fast decay is strongly reduced in the Cs_5_Pb.0.05GuaI film, consistent with the high PL intensity [32].

### 2.2. Morphological and Photovoltaic Characterization

**Figure 2:**
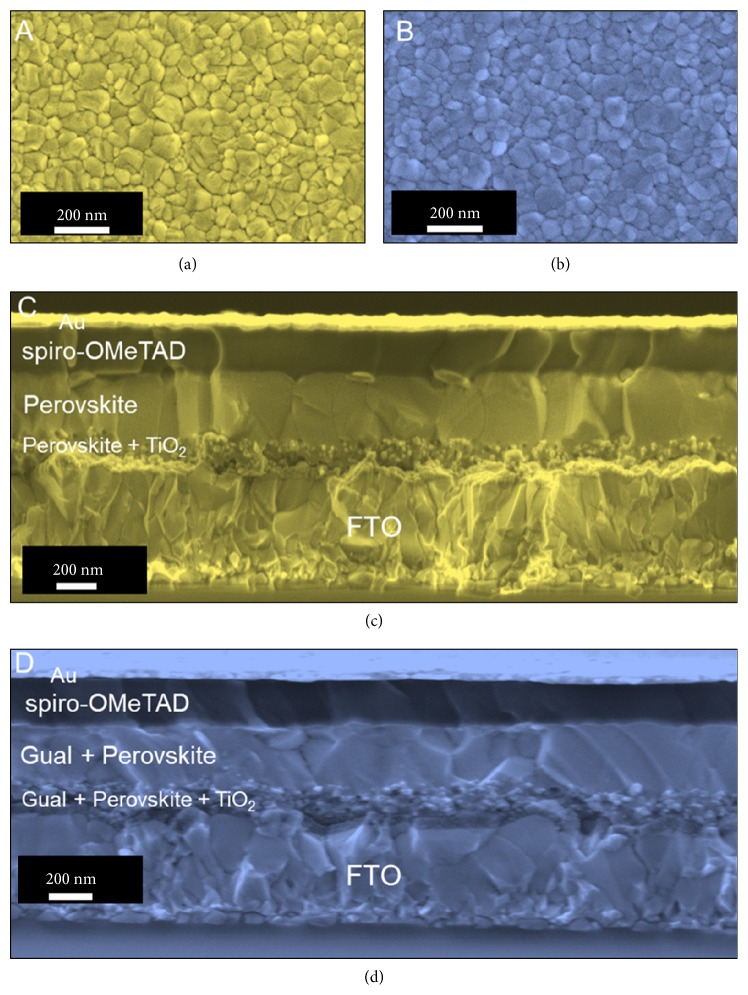
Morphological characterization. (a) and (b) Top view SEM micrographs acquired from Cs_5_Pb, and Cs_5_Pb.0.05GuaI films. (c) and (d) Cross-sectional SEM micrographs acquired from the fully assembled devices based on Cs_5_Pb and Cs_5_Pb.0.05GuaI films.

**Figure 3:**
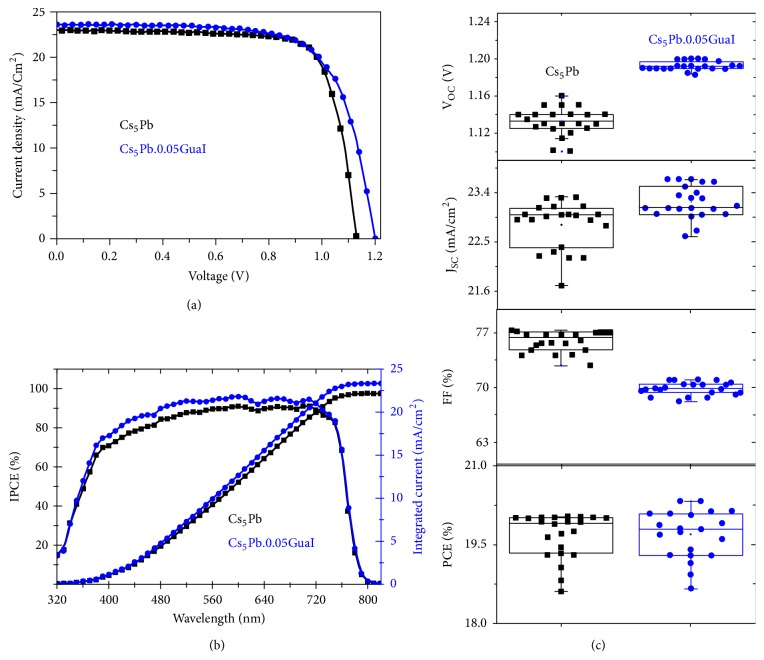
Photovoltaic characteristics of the perovskite solar cells based on Cs_5_Pb and Cs_5_Pb.0.05GuaI films. (a) Current density versus voltage characteristics of perovskite solar cells. (b) Incident photon-to-current efficiency spectra as a function of the wavelength and the corresponding integrated J_SC_. (c) J-V metrics for 22 devices using Cs_5_Pb and Cs_5_Pb·0.05GuaI films.

Furthermore, the surface morphology of perovskite films was characterized via scanning electron microscopy (SEM) (Figure 2). Ostensibly, top view (Figures 2(a) and 2(b)) and cross-sectional (Figures 2(c) and 2(d)) SEM micrographs acquired from Cs_5_Pb, Cs_5_Pb.0.05GuaI perovskite films and the devices based on them confirm that the morphology of absorber layers is not affected by the addition of GuaI. The effect of GuaI incorporation on the photovoltaic performance is further examined in a device configuration of FTO/c-TiO_2_/m-TiO_2_/perovskite/spiro-OMeTAD/Au (for more details see Methods).  Figure 3(a) depicts the* J-V* curves of the devices with 5% GuaI content in comparison to the reference Cs_5_Pb device, and the corresponding photovoltaic parameters are summarized in Table 1. For the Cs_5_Pb device, the highest PCE obtained is 20.03% with an open circuit voltage (*V*_OC_) of 1.13 V, a short-circuit current density (J_SC_) of 23 mA cm^−2^, and a fill factor (FF) of 77%, which is in agreement with the values reported for the cesium-containing triple cation PSCs previously [31].

The PCE of Cs_5_Pb.0.05GuaI based device is comparable to that of the reference device (Table 1). With respect to the Cs_5_Pb based device, the Cs_5_Pb.0.05GuaI devices showed higher J_SC_ values of 23.6 mA/cm^2^. Intriguingly, the addition of 5% GuaI to Cs_5_Pb significantly improved the *V*_OC_ from 1.13 V to 1.20 V. To the best of our knowledge, a *V*_OC_ of 1.20 V is among the highest values reported in the literature for a similarly configured PSC. Consistent with the PL data, Figure 3(b) shows the incident photon-to-current efficiency (IPCE) and integrated current density as a function of wavelength. From the IPCE spectra, we found that upon addition of 5% GuaI (Figure 3(b)), the same number of photons appears to generate more current compared to the reference Cs_5_Pb device.

Based on the photovoltaic characterization, we found that the incorporation of 5% GuaI into mixed-cation and mixed-halide perovskite films improved the overall performance of PSCs. The reproducibility of the device performance was further ascertained by measuring 22 devices each from Cs_5_Pb and Cs_5_Pb.0.05GuaI as shown in Figure 3(c). We noted that a *V*_OC_ between 1.19-1.20 V is consistently achievable. By contrast, the fill factor of the Cs_5_Pb.0.05GuaI devices is lower than that of the Cs_5_Pb reference. Generally, the FF of a solar cell is affected by the series resistance (R_S_) and ideality factor of the device [33]. By taking the slope of light* J-V* characteristics at the *V*_OC_, R_S_ was calculated and summarized in [Supplementary-material supplementary-material-1]. The Cs_5_Pb.0.05GuaI device exhibits a higher value of R_S_ as compared to the Cs_5_Pb device. Therefore, by simply improving the FF, substantially higher PCEs could be realized.

### 2.3. Insight about the Hysteresis Behaviour

**Figure 4:**
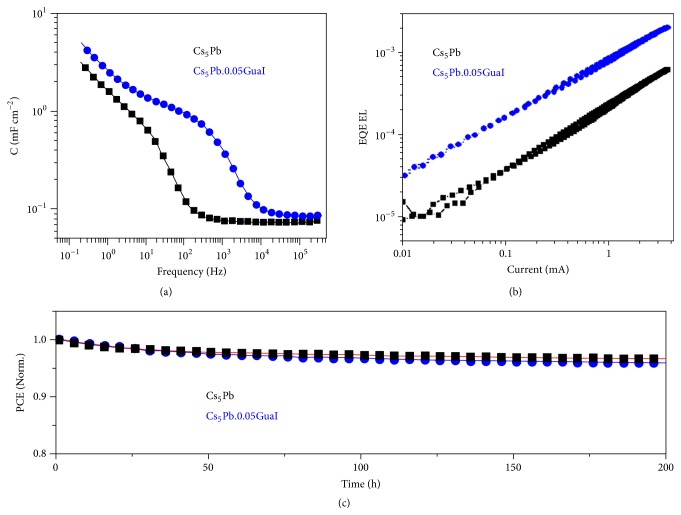
Electroluminescence and stability measurements. (a) Capacitance versus frequency spectra of Cs_5_Pb and Cs_5_Pb.0.05GuaI based devices under short-circuit condition in the frequency range from 200 MHz to 1 MHz. The active area of perovskite solar cell is 0.16 cm^2^ for both measurements. (b) External electroluminescence quantum efficiency as a function of the injection current for the Cs_5_Pb and Cs_5_Pb.0.05GuaI devices. (c) A comparison of operational stability of Cs_5_Pb and Cs_5_Pb.0.05GuaI devices. The devices were measured under a nitrogen environment at room temperature under constant illumination (LED source, approximated 1 Sun) at a maximum power point for 200 h.

The Cs_5_Pb and Cs_5_Pb.0.05GuaI based devices were also examined for hysteresis ([Supplementary-material supplementary-material-1]) and despite favorable properties of Gua ions, such as zero dipole moment, the hysteresis effects were more prominent in GuaI based devices [34]. To understand the cause of the dominant hysteresis feature [35], capacitance-frequency measurements at zero bias in the dark were carried out (Figure 4(a)), where features in the low and high frequency of C-f response are clearly distinguishable. A constant capacitance element at a frequency >10^3^ Hz for Cs_5_Pb and >10^4^ Hz for Cs_5_Pb.0.05GuaI based devices is associated with the dielectric response of the absorber material. In turn, the capacitance in the low-frequency spectra could be associated with the ionic characteristics [35, 36]. Apparently, excess Gua ions, which are not confined within the perovskite crystal lattice and have weak bonding capability, could pile up near the contact interface and screen the local electric field leading to a higher value of capacitance in the low-frequency region [37]. By considering the electrostatic interactions at room temperature, space charge densities of 6.04×10^17^ cm^−3^ and 1.4×10^18^ cm^−3^ were obtained, respectively, for the reference and Cs_5_Pb.0.05GuaI devices. The accumulation of relatively higher ions causes an excess capacitance in the low frequency, which consequently leads to the hysteresis. Furthermore, the motion of these ions under the applied bias can screen the internal electric field at the interface (TiO_2_/perovskite), thus amplifying the hysteresis effect.

### 2.4. Electroluminescence Study

Electroluminescence (EL) measurements were carried out to compare the radiative emission properties of Cs_5_Pb and Cs_5_Pb.0.05GuaI PSCs. The EL was measured during a voltage sweep from 0 to 2 V and back. [Supplementary-material supplementary-material-1] shows the* J-V* curve (solid lines) and emitted photon flux (dashed lines) obtained from Cs_5_Pb and Cs_5_Pb.0.05GuaI devices by a voltage loop starting from 0 V with a scan rate of 20 mV/s. The current onset is shifted towards higher voltages upon addition of GuaI (solid lines), consistent with the increased *V*_OC_. This comes along with a roughly four-times higher radiative emission yield when compared at the same driving current (Figure 4(b)). Therefore, the increased PL signal and lifetime of charge carriers observed in perovskite films could be maintained. Following the approach in [38], *V*_OC_ can be derived from the emission yield (Table 2). The calculated values of 1.16 and 1.19 V for Cs_5_Pb and Cs_5_Pb.0.05GuaI, respectively, coincide with the experimental data.

### 2.5. Operational Stability of the Perovskite Solar Cells

Finally, the operational stability of Cs_5_Pb and Cs_5_Pb.0.05GuaI based devices was investigated at a maximum power point under constant one-sun illumination for 200 h in a nitrogen environment (Figure 4(c)) [39]. It is evident that Cs_5_Pb and Cs_5_Pb.0.05GuaI remain relatively stable for almost 200 h, which indicates that the incorporation of Gua cations, an organic species, does not introduce any additional source of degradation [40]. Thus far, such a promising operational stability has not been demonstrated for >20% efficient guanidinium-based PSCs yielding photovoltage as high as 1.20 V, which makes this investigation highly important.

## 3. Discussion

We investigated the effect of guanidinium iodide incorporation into mixed-cation and mixed-halides perovskite films. Apparently, a slight red shift from 757 to 766 nm in the PL peak position indicates the incorporation of GuaI in the perovskite lattice. A gradual increase in the content of GuaI shows a significant enhancement of the open circuit voltage from 1.13 V to 1.20 V leading to the realization of >20.3% PCE. The increased photoluminescence and lifetime of charge carriers observed in GuaI containing perovskite films were maintained in the fully assembled device, justifying the trends in the *V*_OC_. PSCs containing a definite amount of GuaI showed four-times higher radiative emission yield than Cs_5_Pb devices. The accumulation of higher ions in GuaI based PSCs causes an excess capacitance in the low-frequency response of capacitance-frequency measurements, which eventually increased the hysteresis. The realization of high photovoltages is quite intriguing, although while dealing with low FF values. Both parameters, i.e., V_OC_ and FF, are interdepended as both are subservient to the ideality factor. Therefore, by simply improving the electronic and interfacial quality in the guanidinium iodide based perovskite systems, the FF values could be improved. Consequently, much higher photovoltages thus efficiencies exceeding record values can be achieved from the resulting perovskite solar cells. Finally, the thorough understanding gained through in-depth analyses unfolded the reasons leading to the realization of operationally stable and highly efficient (>20%) PSCs yielding photovoltage as high as 1.20 V. Our study shows that there is still plenty of room to improve PCE to new record levels by strategically manoeuvring the precursor chemistry.

## 4. Materials and Methods

### 4.1. Experimental Design

We aimed to fabricate highly reproducible and highly efficient perovskite solar cell yielding remarkable photovoltages by judiciously tailoring the photophysical properties of perovskite structures. In this direction, the following steps and detailed characterization techniques, including spectroscopy, scanning electron microscopy, X-ray diffraction, and other device characterization techniques, were employed.

### 4.2. Materials

All materials were purchased from Sigma-Aldrich and were used as received, unless stated otherwise.

### 4.3. Substrate Preparation

Fluorine-doped tin oxide (FTO)-glass substrates (TCO glass, NSG 10, Nippon sheet glass, Japan) were chemically etched using Zn powder and 4 M HCl and cleaned by ultrasonication in Hellmanex (2%, deionized water), rinsed thoroughly with deionized water and ethanol, and then treated with oxygen plasma for 15 min. A 30 nm blocking layer (TiO_2_) was deposited on the cleaned FTO by spray pyrolysis at 450°C using a commercial titanium diisopropoxide bis(acetylacetonate) solution (75% in 2-propanol, Sigma-Aldrich) diluted in anhydrous ethanol (1:9, volume ratio) as precursor and oxygen as a carrier gas. A 150 nm mesoporous TiO_2_ layer was deposited by spin coating a diluted paste (1:6 wt. ratio) (Dyesol 30NRD: ethanol) (4000 rpm, acceleration 2000 rpm for 20 s) onto the substrate containing TiO_2_ compact layer and then sintered at 450°C for 30 min in dry air. For Li treatment of mesoporous TiO_2_, 150 *μ*L of LiTFSI solution in acetonitrile (10mg/mL, freshly prepared in an argon atmosphere) was spin coated (3000 rpm, acceleration 2000 rpm for 20 s) after a loading time of 10 s. Thereafter, Li treated substrates were sintered at 450°C for 30 min.

### 4.4. Deposition of Perovskite Films

The perovskite films were deposited using a single-step deposition method from the precursor solution containing FAI (1M), PbI_2_ (1.1 M), MABr (0.2 M), and PbBr_2_ (0.2 M) in anhydrous dimethylformamide/ dimethylsulphoxide (4:1 (volume ratio)). Thereafter, 5% of CsI (Acros 99.9%) (1.5 M DMSO) (1.5 M DMSO) was added to the perovskite precursor solution. The precursor solution was spin coated onto the mesoporous TiO_2_ films in a two-step program at 1000 and 6000 r.p.m. for 10 and 30 s, respectively. During the second step, 130 *μ*l of chlorobenzene was dropped on the spinning substrate 10 s prior the end of the program. This was followed by annealing the films at 100°C for 40 min. The device fabrication was carried out under controlled atmospheric conditions with humidity <2%. For completing the fabrication of devices, 2,2',7,7'-tetrakis(N,N-di-p-methoxyphenylamine)-9,9-spirobifluorene (spiro-OMeTAD, 60 mM in chlorobenzene), the HTM was doped with bis(trifluoromethylsulfonyl)imide lithium salt, tris(2-(1H-pyrazol-1-yl)-4-tert-butylpyridine)- cobalt(III) tris(bis(trifluoromethylsulfonyl) imide) (FK 209, from Dyenamo), and 4-tert-Butylpyridine in a molar ratios of 0.5, 0.05, and 3.3, respectively. Finally, ~75 nm gold (Au) layer was thermally evaporated.

### 4.5. Device Characterization

The current-voltage (J-V) characteristics of the perovskite devices were recorded with a digital source meter (Keithley model 2400, USA). A 450 W xenon lamp (Oriel, USA) was used as the light source for photovoltaic (J-V) measurements. The spectral output of the lamp was filtered using a Schott K113 Tempax sunlight filter (Präzisions Glas & Optik GmbH, Germany) to reduce the mismatch between the simulated and actual solar spectrum to less than 2%. The photo-active area of 0.16 cm^2^ was defined using a dark-coloured metal mask. The IPCE measurements were performed using a LED light source (Ariadne EQE from Cicci Research).

### 4.6. Morphological Characterization

Scanning electron microscopy (SEM) was performed on a ZEISS Merlin HR-SEM using an In-lens detector.

### 4.7. Structural Characterization

X-ray diffraction data were collected on a Bruker Advance D8 X-ray diffractometer with a graphite monochromator, using Cu K*α* radiation.

### 4.8. Spectroscopic Measurements

UV–vis measurements were performed on a Varian Cary 5. Photoluminescence spectra were obtained with a Florolog 322 (Horiba Jobin Ybon Ltd) in the wavelength range from 500 nm to 850 nm by exciting at 460 nm. The spectrometer working in a time-correlated single-photon counting mode with <ns time resolution was used for the time-resolved photoluminescence studies. Picosecond pulsed diode laser head NanoLED-405LH (Horiba) emitting <200 ps duration pulses at 408 nm with a repetition rate of 1 MHz was used as an excitation source.

### 4.9. Electroluminescence Measurements

The emitted photon flux was detected with a large-area (1 cm^2^) Si-photodiode (Hamamatsu S1227-1010BQ) positioned close to the sample. The voltage scan was performed using a Bio-Logic SP300 potentiostat, which was also used to simultaneously measure the short-circuit current of the photodiode connected to a second channel.

### 4.10. Long-Term Light Soaking Test

Stability measurements were performed using a home-built system with white LED illumination with an intensity equivalent to 1 sun. The devices were kept at maximum power point (MPP) by a custom-made computer controlled MPP tracking routine. The inert atmosphere was achieved by flushing the sample holder with nitrogen.

## Figures and Tables

**Table 1:** Summarized J-V characteristics. J-V characteristics of the best devices with 5% GuaI content in comparison to the reference Cs_5_Pb device.

Samples	*J*_SC_ (mA/cm^2^)	*V*_OC_ (V)	FF (%)	PCE (%)
Cs_5_Pb	23.0	1.13	77	20.0
Cs_5_Pb.0.05GuaI	23.6	1.20	70	20.3

**Table 2:** Summarized electroluminescence data. Summary of the observed and calculated parameters derived from the electroluminescence data.

Device	*V*_OC_, rad	EQE EL at injection current approx. *J*_SC_	Nonrad. loss	*V*_OC_, calc.
Cs_5_Pb	1.34 V	0.09 %	180 mV	1.16 V
Cs_5_Pb.0.05GuaI	1.34 V	0.3 %	150 mV	1.19 V

## Supplementary Materials

Supplementary Materials Figure S1. Photoluminescence spectra recorded from Cs_5_Pb.*x*GuaI films deposited onto the mesoporous Al_2_O_3_ substrate. Table S1. Summarized* J-V* characteristic of the devices with 5% GuaI content in comparison to the reference Cs_5_Pb device. Figure S2. Forward and reverse current-voltage scans for (a) Cs_5_Pb and (b) Cs_5_Pb.0.05GuaI devices. Figure S3.* J-V* curve (solid lines) and emitted photon flux (dashed lines) obtained from Cs_5_Pb and Cs_5_Pb.0.05GuaI devices by a voltage loop starting from 0 V with a scan rate of 20 mV/s. Differences in current at low voltages are due to the hysteresis phenomenon. Figure S4. X-ray diffraction patterns corresponding to pure and GuaI containing perovskite films.
