# Supplementary material for: Perovskite Solar Cells Yielding Reproducible Photovoltage of 1.20 V
Source: Research (Wash D C). 2019 Mar 18;2019:8474698. doi: 10.34133/2019/8474698 (PMC6750062; doi:10.34133/2019/8474698)
Supplement: Supplementary Materials — Figure S1. Photoluminescence spectra recorded from Cs5Pb.xGuaI films deposited onto the mesoporous Al2O3 substrate. Table S1. Summarized J-V characteristic of the devices with 5% GuaI content in comparison to the reference Cs5Pb device. Figure S2. Forward and reverse current-voltage scans for (a) Cs5Pb and (b) Cs5Pb.0.05GuaI devices. Figure S3. J-V curve (solid lines) and emitted photon flux (dashed lines) obtained from Cs5Pb and Cs5Pb.0.05GuaI devices by a voltage loop starting from 0 V with a scan rate of 20 mV/s. Differences in current at low voltages are due to the hysteresis phenomenon. Figure S4. X-ray diffraction patterns corresponding to pure and GuaI containing perovskite films. [file 8474698.f1.docx]

Supplementary Materials

**Fig. S1**. Photoluminescence spectra recorded from Cs_5_Pb.*x*GuaI films deposited onto the mesoporous Al_2_O_3_ substrate.

| Samples | *J*_SC_(mA/cm^2^) | *V*_OC_ (V) | FF (%) | PCE (%) | Series Resistance  (R_s_) (Ω.cm^2^) |
| --- | --- | --- | --- | --- | --- |
| Cs_5_Pb | 23.0 | 1.13 | 76.8 | 20.0 | 3.5 |
| Cs_5_Pb.0.05GuaI | 23.6 | 1.20 | 70.3 | 20.3 | 5.0 |

**Table S1.** Summarized *J-V* characteristic of the devices with 5% GuaI content in comparison to the reference Cs_5_Pb device:

**
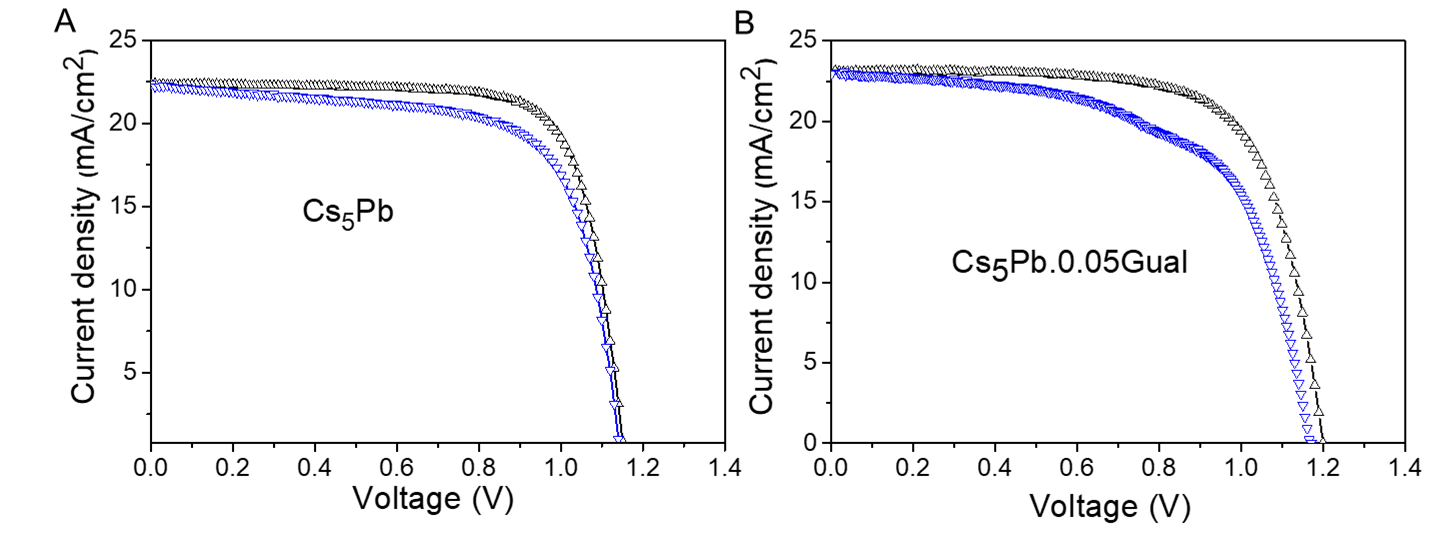
**

**Fig. S2**. Forward and reverse current-voltage scans for (a) Cs_5_Pb, and (b) Cs_5_Pb.0.05GuaI devices.


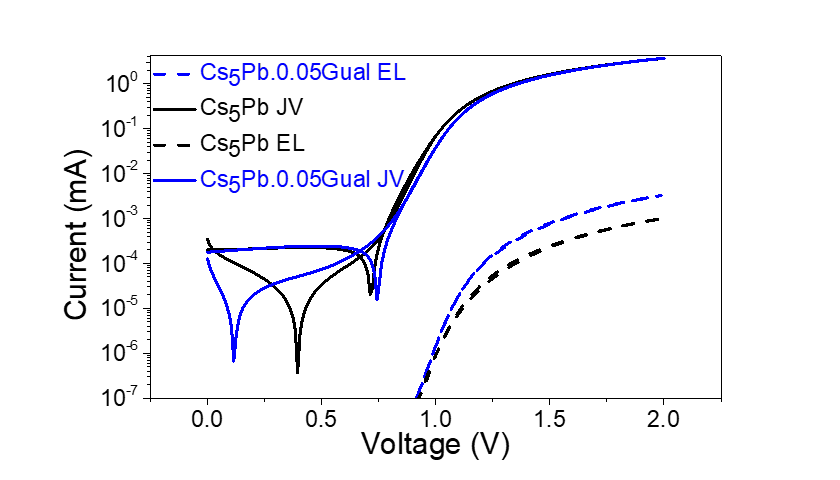


**Fig. S3**. *J-V* curve (solid lines) and emitted photon flux (dashed lines) obtained from Cs_5_Pb and Cs_5_Pb.0.05GuaI devices by a voltage loop starting from 0 V with a scan rate of 20 mV/s. Differences in current at low voltages are due to the hysteresis phenomenon.

**Fig. S4**. X-ray diffraction patterns corresponding to pure and GuaI containing perovskite films.
